# Cell-autonomous reduction of CYFIP2 is insufficient to induce Alzheimer's disease-like pathologies in the hippocampal CA1 pyramidal neurons of aged mice

**DOI:** 10.1080/19768354.2023.2192263

**Published:** 2023-03-24

**Authors:** Ruiying Ma, Yinhua Zhang, Huiling Li, Hyae Rim Kang, Yoonhee Kim, Kihoon Han

**Affiliations:** a Department of Neuroscience, Korea University College of Medicine, Seoul, Republic of Korea; b BK21 Graduate Program, Department of Biomedical Sciences, Korea University College of Medicine, Seoul, Republic of Korea

**Keywords:** CYFIP2, Alzheimer’s disease, hippocampal CA1, excitatory pyramidal neuron, conditional knock-out

## Abstract

Cytoplasmic FMR1-interacting protein 2 (CYFIP2) is an evolutionarily conserved multifunctional protein that regulates the neuronal actin cytoskeleton, mRNA translation and transport, and mitochondrial morphology and function. Supporting its critical roles in proper neuronal development and function, human genetic studies have repeatedly identified variants of the *CYFIP2* gene in individuals diagnosed with neurodevelopmental disorders. Notably, a few recent studies have also suggested a mechanistic link between reduced CYFIP2 level and Alzheimer's disease (AD). Specifically, in the hippocampus of 12-month-old *Cyfip2* heterozygous mice, several AD-like pathologies were identified, including increased levels of Tau phosphorylation and gliosis, and loss of dendritic spines in CA1 pyramidal neurons. However, detailed pathogenic mechanisms, such as cell types and their circuits where the pathologies originate, remain unknown for AD-like pathologies caused by CYFIP2 reduction. In this study, we aimed to address this issue by examining whether the cell-autonomous reduction of CYFIP2 in CA1 excitatory pyramidal neurons is sufficient to induce AD-like phenotypes in the hippocampus. We performed immunohistochemical, morphological, and biochemical analyses in 12-month-old *Cyfip2* conditional knock-out mice, which have postnatally reduced CYFIP2 expression level in CA1, but not in CA3, excitatory pyramidal neurons of the hippocampus. Unexpectedly, we could not find any significant AD-like phenotype, suggesting that the CA1 excitatory neuron-specific reduction of CYFIP2 level is insufficient to lead to AD-like pathologies in the hippocampus. Therefore, we propose that CYFIP2 reduction in other neurons and/or their synaptic connections with CA1 pyramidal neurons may be critically involved in the hippocampal AD-like phenotypes of *Cyfip2* heterozygous mice.

## Introduction

The two members of the cytoplasmic FMR1-interacting protein family, CYFIP1 and CYFIP2, are evolutionarily conserved proteins whose genetic variants are causally associated with numerous brain disorders, including autism spectrum disorders, intellectual disability, schizophrenia, and epilepsy (Schenck et al. 2001; Abekhoukh and Bardoni 2014; Zhang, Lee, et al. 2019). Specifically, in the case of *CYFIP2*, *de novo* variants have recently been identified in individuals diagnosed with neurodevelopmental disorders and early-onset epileptic encephalopathy characterized by developmental regression, intellectual disability, seizures, muscular hypotonia, and microcephaly (Nakashima et al. 2018; Peng et al. 2018; Lee et al. 2019; Zhong et al. 2019; Zweier et al. 2019; Begemann et al. 2021; Kang et al. 2023).

At the molecular level, CYFIP1 and CYFIP2 have a high amino acid sequence homology and both are involved in the regulation of cellular actin cytoskeleton dynamics as a critical component of the heteropentameric Wiskott–Aldrich syndrome protein family verprolin-homologous protein (WAVE) regulatory complex (WRC) (Lee Y et al. 2017; Rottner et al. 2021). Additional functions in neurons, such as regulation of mRNA translation and transport (Napoli et al. 2008; De Rubeis et al. 2013; Cioni et al. 2018), and regulation of mitochondrial function and morphology (Kanellopoulos et al. 2020; Kim GH et al. 2020) have also been identified, but unlike actin regulation via the WRC, these functions may be mediated by both shared and distinct interactors of CYFIP1 and CYFIP2, respectively (Lee Y et al. 2020; Ma et al. 2022). Collectively, these molecular functions of CYFIP1 and CYFIP2 can contribute to the morphological and functional changes in neuronal synapses observed in both *Cyfip1* and *Cyfip2* mutant mice (Bozdagi et al. 2012; Pathania et al. 2014; Han et al. 2015; Davenport et al. 2019; Lee SH et al. 2020; Zhang et al. 2020; Kim NS et al. 2022).

Moreover, several lines of evidence indicate the differential roles of CYFIP1 and CYFIP2 *in vivo*, including the embryonic and perinatal lethality of *Cyfip1*-null mice and *Cyfip2*-null mice, respectively (Chung et al. 2015; Han et al. 2015; Zhang et al. 2019a), and different brain regional and cell-type distributions of CYFIP1 and CYFIP2 mRNAs and proteins (Zhang, Kang, et al. 2019b; Ma et al. 2022). In particular, CYFIP1, but not CYFIP2, is also expressed in non-neuronal cells, such as astrocytes, microglia, and oligodendrocytes, in the brain (Dominguez-Iturza et al. 2019; Silva et al. 2019; Habela et al. 2020; Haan et al. 2021; Ma et al. 2022).

Beyond the genetic association between *CYFIP2* and neurodevelopmental disorders, recent studies have suggested a mechanistic association between the reduction of CYFIP2 and Alzheimer's disease (AD) (Tiwari et al. 2016; Ghosh et al. 2020). Specifically, CYFIP2 protein levels were reduced in the postmortem forebrain of patients with AD and in the hippocampus and cortex of AD model mice (Tiwari et al. 2016). Moreover, the protein levels of amyloid precursor protein (APP), β-site APP cleaving enzyme 1 (BACE1), and calcium/calmodulin-dependent protein kinase IIα (CaMKIIα) were up-regulated in the hippocampal synaptosomal fraction of conventional *Cyfip2* heterozygous (*Cyfip2^+/–^*, het) mice. Consistently, in the hippocampus of aged (12-month-old) *Cyfip2* het mice, several AD-like pathologies have been observed, including increased levels of Tau phosphorylation and gliosis, and significant loss of dendritic spines in CA1 pyramidal neurons (Ghosh et al. 2020). Mechanistically, it has been proposed that reduced expression level of CYFIP2 induces aberrant local mRNA translation of several AD-related proteins (i.e. APP, BACE1, and CaMKIIα) at the synaptic compartment, thereby leading to the overproduction of Aβ and hyperphosphorylation of Tau in *Cyfip2* het mice (Ghosh et al. 2020). Under normal conditions, CYFIP2, as shown for CYFIP1 (Napoli et al. 2008; De Rubeis et al. 2013), may repress the translation of these mRNAs by forming an inhibitory complex with the RNA-binding protein fragile X messenger ribonucleoprotein (FMRP) and the eukaryotic initiation factor 4E (eIF4E).

However, compared to CYFIP2 function and dysfunction in the developing brain, its role in AD-like pathologies in aged mice remains largely unknown. In particular, considering the complex interactions between different neuronal and non-neuronal cell types and the trans-synaptic spread of pathology in AD (Tzioras et al. 2023), investigating the cell types and their circuits where CYFIP2-dependent pathologies originate is a critical step toward understanding the pathogenic mechanisms. In this study, we aimed to address this issue by examining the AD-like phenotypes of aged (12-month-old) *Cyfip2* conditional knock-out (cKO) mice, which have postnatally reduced CYFIP2 expression level selectively in CA1, but not in CA3, excitatory pyramidal neurons of the hippocampus. Surprisingly, unlike *Cyfip2* het mice, there was no overt AD-like phenotype in the hippocampal CA1 region of aged *Cyfip2* cKO mice. Therefore, our results suggest that cell-autonomous reduction of CYFIP2 is insufficient for AD-like pathologies in CA1 pyramidal neurons and that other neurons and/or their synaptic connections with CA1 pyramidal neurons are also critically involved in the hippocampal AD-like phenotypes of *Cyfip2* het mice.

## Materials and methods

### Mice

The *Cyfip2* cKO mice and *Thy1-YFP* mice used in this study were previously described (Lee SH et al. 2020; Zhang et al. 2020). The mice were fed ad libitum and housed under a 12 h light–dark cycle. All experiments were performed using aged (12-month-old) male *Cyfip2* cKO mice and their littermate controls.

### Fluorescence immunohistochemistry

Fluorescence immunohistochemistry was performed as previously described (Lee B et al. 2017; Yu et al. 2021). Mice were anesthetized with isoflurane and transcardially perfused with heparinized (20 units/mL) phosphate-buffered saline (PBS), followed by 4% paraformaldehyde (PFA) in PBS. The brains were extracted and post-fixed overnight in 4% PFA. Following post-fixation, brain tissue was washed with PBS and cryoprotected with 30% sucrose in PBS for 48 h. The brain tissues were frozen in an O.C.T compound (SAKURA Tissue-Tek, 4583) and sectioned (60 µm) using a cryostat microtome (Leica, CM3050S). The primary antibodies used for immunohistochemistry were AT-8 (Phospho-Tau [Ser202, Thr205], Invitrogen, #MN1020), CYFIP1 (Sigma-Aldrich, #AB6046), CYFIP2 (Abcam, #ab95969), GFAP (Abcam, #ab4674), Iba1 (Synaptic System, #234-006), NeuN (Abcam, #ab177487; Millipore, #MAB377). F-actin was visualized by Alexa Fluor 488-conjugated Phalloidin (Invitrogen, #A-12379). The samples were washed with 0.1% Triton X-100 in PBS and blocked with PBS containing 3% bovine serum albumin (BSA) and 0.5% Triton X-100. The high-resolution image acquisition was performed using a Zeiss LSM800 confocal microscope equipped with a 20×/0.8 objective lens, 8-bit image depth, and snapshot mode focused on maximum intensity. Whole brain regions were obtained using a slide scanner (Zeiss Axio Scan.Z1). The regions of the stratum oriens (SO) and stratum radiatum (SR) were defined as 100 µm and 100–200 µm away from the cell body area (stratum pyramidale, SP) of CA1, respectively. The values of at least two brain sections were averaged for each mouse.

### Dendritic spine analysis

The dendritic spine analysis was performed as previously described (Han et al. 2013; Choi et al. 2015; Hong et al. 2022). Mice were deeply anesthetized with isoflurane and transcardially perfused with heparinized (20 units/mL) PBS followed by 4% PFA in PBS. The brains were extracted and post-fixed overnight in 4% PFA. After post-fixation, coronal sections (100 µm thickness) of the hippocampal region were obtained using a vibratome (VT1000S, Leica). The sections were collected and stored in 50% glycerol in 2 × PBS at −20^◦^C until further processed. Blocking, permeabilization, and anti-GFP (Abcam, #AB13970) primary and Alexa Fluorconjugated (anti-chicken Alexa Fluor-488, Jackson ImmunoResearch Labs, #703-545-155) secondary antibody incubation were performed as described above. Finally, the sections were mounted on slide glasses with mounting media (Biomeda, M02). Images of dendritic spines in the secondary or tertiary branches (apical or basal dendrites of YFP-positive CA1 pyramidal neurons in the hippocampus) were acquired by confocal microscopy (Zeiss LSM800) using 63×/1.2 water immersion objective lens, 8-bit image depth, and Z-stack function with 0.93 µm intervals, followed by Z-stack projection of maximum intensity. Images were analyzed using ImageJ software. For quantification of dendritic spines, mushroom spines were defined as protrusions with heads and with a width greater than length. Stubby spines were defined as protrusions without a neck. The rest of the protrusions with heads were categorized as thin spines. The values of six to eight neurons were averaged for each mouse.

Additional information of Materials and Methods is included in supporting online material.

## Results

### CA1 excitatory pyramidal neuron-specific reduction of CYFIP2 in the hippocampus of aged Cyfip2 cKO mice

**Figure 1.**
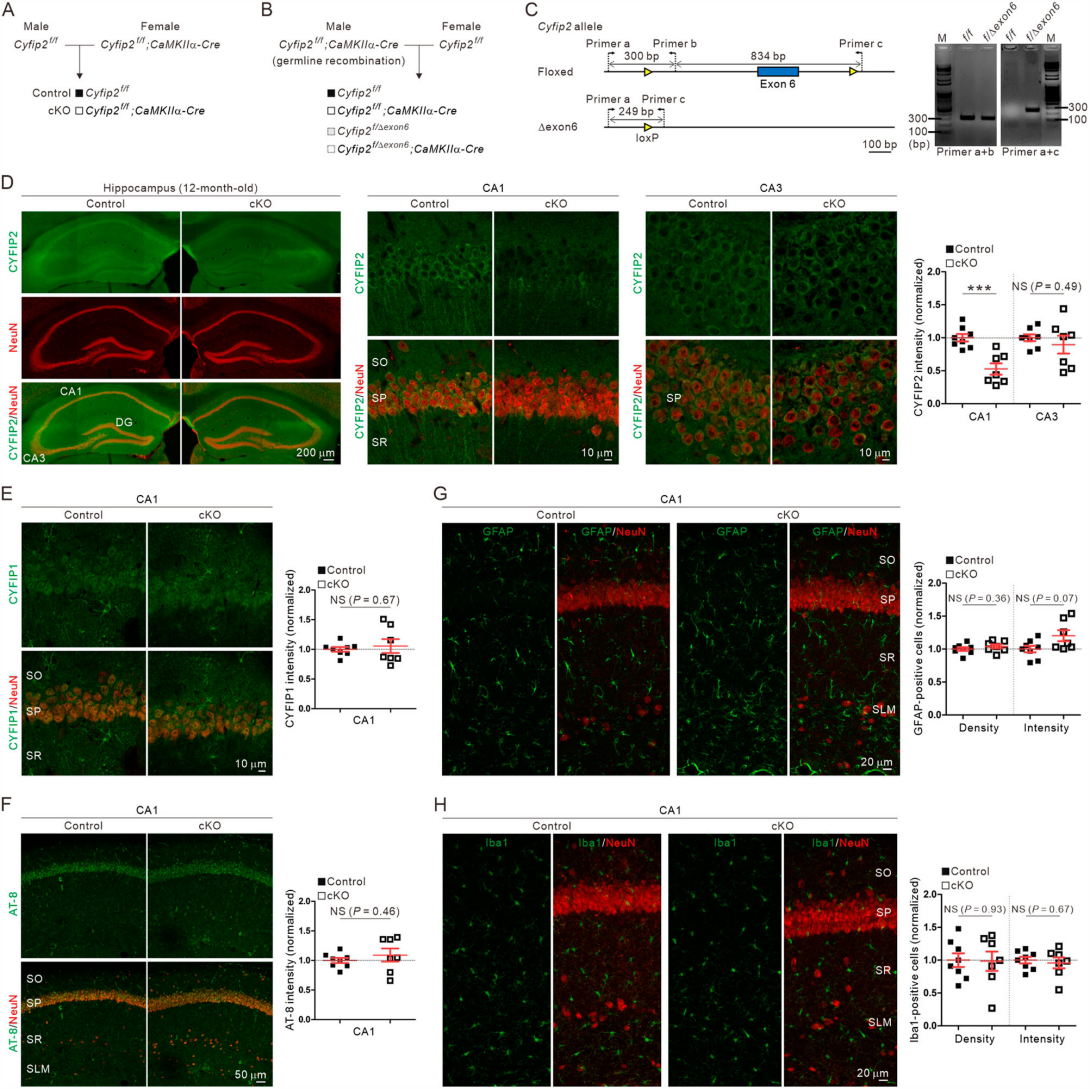
No AD-like immunohistological phenotype in the hippocampal CA1 region of aged *Cyfip2* cKO mice. (A) The breeding scheme for the control (*Cyfip2^f/f^*) and *Cyfip2* conditional knock-out (*Cyfip2^f/f^;CaMKIIα-Cre*, cKO) mice. (B) The breeding scheme to test the partial germline recombination of male *CaMKIIα-Cre* mice. (C) Design of primers to detect floxed and Δexon6 *Cyfip2* alleles (left panel). Results of PCR for the tail genomic DNA isolated from *Cyfip2^f/f^* and *Cyfip2^floxed/Δexon6^* mice (right panel). Note that the primer set (a + c) does not produce the expected ∼1.1 kbp band from the DNA sample of *Cyfip2^f/f^* mice due to the short elongation time of the PCR. (D) Fluorescence immunohistochemistry images and quantification showing CA1-specific reduction of CYFIP2 in the hippocampus of aged *Cyfip2* cKO mice. CA, cornu ammonis; DG, dentate gyrus; NS, not significant; SO, stratum oriens; SP, stratum pyramidale; SR, stratum radiatum. (E, F) Normal CYFIP1 and phospho-Tau (AT-8) levels in the hippocampal CA1 region of aged *Cyfip2* cKO mice. (G, H) Normal density and total intensity of astrocytes (GFAP-positive) and microglia (Iba1-positive) in the hippocampal CA1 region of aged *Cyfip2* cKO mice. *N* = 7–8 mice.

To investigate whether cell-autonomous CYFIP2 reduction in excitatory pyramidal neurons is sufficient to induce AD-like phenotypes in the hippocampal CA1 region, we crossed *floxed-Cyfip2* mice with *CaMKIIα-Cre* mice to generate *Cyfip2* cKO (*Cyfip2^floxed/floxed^*; *CaMKIIα-Cre*) mice as previously described (Zhang et al. 2020). The *CaMKIIα-Cre* line (T29-1) used in this study starts expressing Cre recombinases in forebrain excitatory neurons during the third to fourth postnatal weeks, and especially in the hippocampus, Cre expression is restricted mainly to the CA1 region (Tsien et al. 1996). Notably, to securely obtain control (*Cyfip2^floxed/floxed^*) and *Cyfip2* cKO progeny mice, we crossed male *Cyfip2^floxed/floxed^* mice with female *Cyfip2^floxed/floxed^;CaMKIIα-Cre* mice (Figure 1(A)) to avoid unwanted germline recombination, which was recently reported in male, but not female, T29-1 mice (Luo et al. 2020). Furthermore, we designed an additional primer set for genotyping PCR to detect germline deletion of floxed exon 6 of the *Cyfip2* gene (Lee SH et al. 2020), which indeed produced an expected PCR band from some portions of progeny mice (i.e. *Cyfip2^floxed/Δexon6^* mice) when we crossed male *Cyfip2^floxed/floxed^;CaMKIIα-Cre* mice with female *Cyfip2^floxed/floxed^* mice as a test (Figure 1(B) and (C)). Using this primer set, we confirmed that the control and *Cyfip2* cKO mice used in this study did not have a germline deletion of the floxed *Cyfip2* exon 6.

Fluorescence immunohistochemical analysis showed that CYFIP2 protein levels were selectively reduced in the hippocampal CA1, but not CA3, of 12-month-old *Cyfip2* cKO mice (Figure 1(D)), which was expected from CA1-restricted Cre expression in T29-1 mice. Meanwhile, CYFIP1 protein levels in the CA1 region were comparable between control and *Cyfip2* cKO mice, suggesting that there is no compensatory increase in CYFIP1 level in the hippocampus of aged *Cyfip2* cKO mice (Figure 1(E)).

### No AD-like immunohistological phenotype in the hippocampal CA1 region of aged Cyfip2 cKO mice

A previous study showed that several AD-like immunohistological phenotypes were significantly exacerbated in the hippocampal CA1 region of 12-month-old *Cyfip2* het mice compared with age-matched wild-type (WT) mice (Ghosh et al. 2020). These phenotypes include increased levels of phospho-Tau immunoreactivity (measured by monoclonal AT-8 antibody) and gliosis (both for astrocytes and microglia, as measured by glial fibrillary acidic protein [GFAP] and ionized calcium-binding adapter molecule 1 [Iba1] antibodies, respectively). Therefore, we performed fluorescence immunohistochemical analyses of these AD markers in the hippocampal CA1 region of 12-month-old *Cyfip2* cKO mice and their littermate controls. However, there were no significant differences in phospho-Tau levels between control and *Cyfip2* cKO mice (Figure 1(F)). Moreover, neither GFAP nor Iba1 positive cell number or total intensity was significantly altered in *Cyfip2* cKO mice compared to control mice (Figure 1(G) and (H)).

### Reduced number of immature dendritic spines and increased F-actin levels in the basal dendrites of CA1 pyramidal neurons of aged Cyfip2 cKO mice

**Figure 2.**
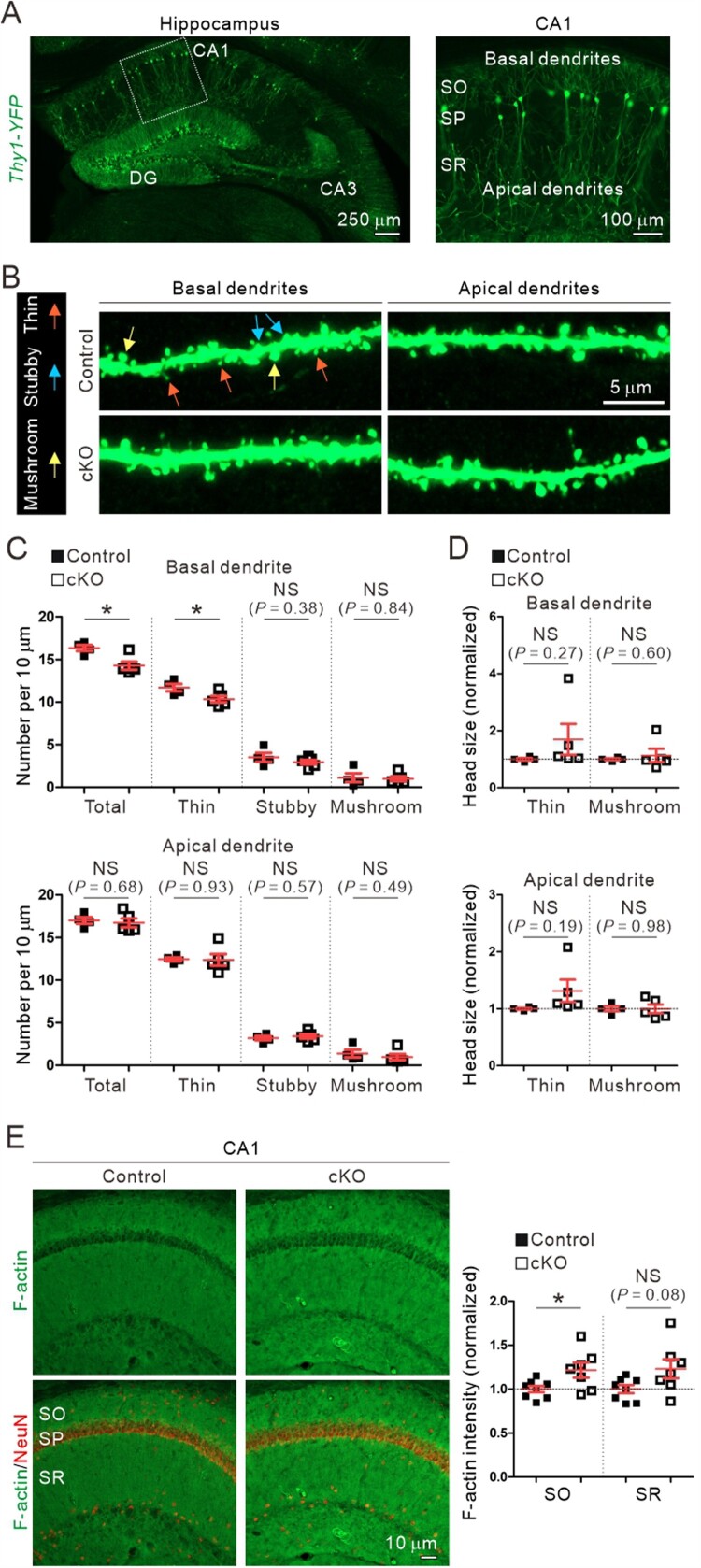
Dendritic spine and F-actin changes in the hippocampal CA1 region of aged *Cyfip2* cKO mice. (A) Visualization of CA1 pyramidal neurons in *Thy1-YFP* mice by sparse expression of yellow fluorescent protein (YFP). CA, cornu ammonis; DG, dentate gyrus; SO, stratum oriens; SP, stratum pyramidale; SR, stratum radiatum. (B) Representative confocal images of dendritic spines in the basal and apical dendrites of CA1 pyramidal neurons of aged control and *Cyfip2* cKO mice. Examples of dendritic spines in each morphologically-based categorization (thin, stubby, and mushroom) are indicated by arrows with different colors. (C) Quantification of dendritic spine number in the basal (upper panel) and apical (lower panel) dendrites. NS, not significant. (D) Quantification of dendritic spine head size in basal (upper panel) and apical (lower panel) dendrites. (E) Representative confocal images and quantification of F-actin levels in the hippocampal CA1 region of aged control and *Cyfip2* cKO mice. *N* = 4–8 mice.

Dendritic spines are small dendritic protrusions that represent the most excitatory postsynapses in the brain (Penzes et al. 2011). Loss of dendritic spines is another key feature of AD (Dorostkar et al. 2015) and was significantly aggravated in CA1 pyramidal neurons of 12-month-old *Cyfip2* het mice compared to age-matched WT mice (Ghosh et al. 2020). In particular, the number of mature-type mushroom spines, but not that of immature-type thin spines, was reduced in the apical dendrites of CA1 pyramidal neurons in aged *Cyfip2* het mice. Therefore, we analyzed the dendritic spines of CA1 pyramidal neurons in 12-month-old control and *Cyfip2* cKO mice. Dendritic spines were visualized by crossing *Cyfip2* cKO mice with *Thy1-YFP* mice (Feng et al. 2000) that sparsely express yellow fluorescent protein (YFP) in CA1 pyramidal neurons (Figure 2(A)). We separately analyzed the basal and apical dendrites of the neurons (Figure 2(B)). In the basal dendrites, we found that the total number of dendritic spines was significantly reduced in *Cyfip2* cKO neurons compared to control neurons (Figure 2(C)). However, unlike the mushroom spine-specific reduction in *Cyfip2* het mice (Ghosh et al. 2020), the spine reduction in *Cyfip2* cKO mice was mainly attributed to a decrease in immature-type thin spines. Moreover, in the apical dendrites, neither total density nor morphologically-based categorization of dendritic spines was altered in *Cyfip2* cKO neurons compared to control neurons (Figure 2(C)), suggesting that there is no AD-like dendritic spine phenotype in CA1 pyramidal neurons of aged *Cyfip2* cKO mice. We also compared head size for both thin and mushroom spines between the control and *Cyfip2* cKO neurons and found no significant differences in the basal or apical dendrites (Figure 2(D)).

F-actin is a key cytoskeletal component of dendritic spines and is directly associated with their formation, maintenance, and dynamics (Spence and Soderling 2015). As a critical component of the WRC, CYFIP2 is involved in actin regulation in various cellular compartments, including neuronal dendritic spines (Rottner et al. 2021). Specifically, it has been previously shown increased F-actin levels in the medial prefrontal cortex (mPFC) of young adult *Cyfip2* het and *Cyfip2* cKO mice (Lee SH et al. 2020; Zhang et al. 2020). Therefore, we also measured F-actin levels in the hippocampal CA1 region and found an increase in the stratum oriens (SO), but not in the stratum radiatum (SR), of aged *Cyfip2* cKO mice compared to control mice (Figure 2(E)).

### Normal expression of synaptosomal APP and CaMKIIα in the hippocampal CA1 region of aged Cyfip2 cKO mice

**Figure 3.**
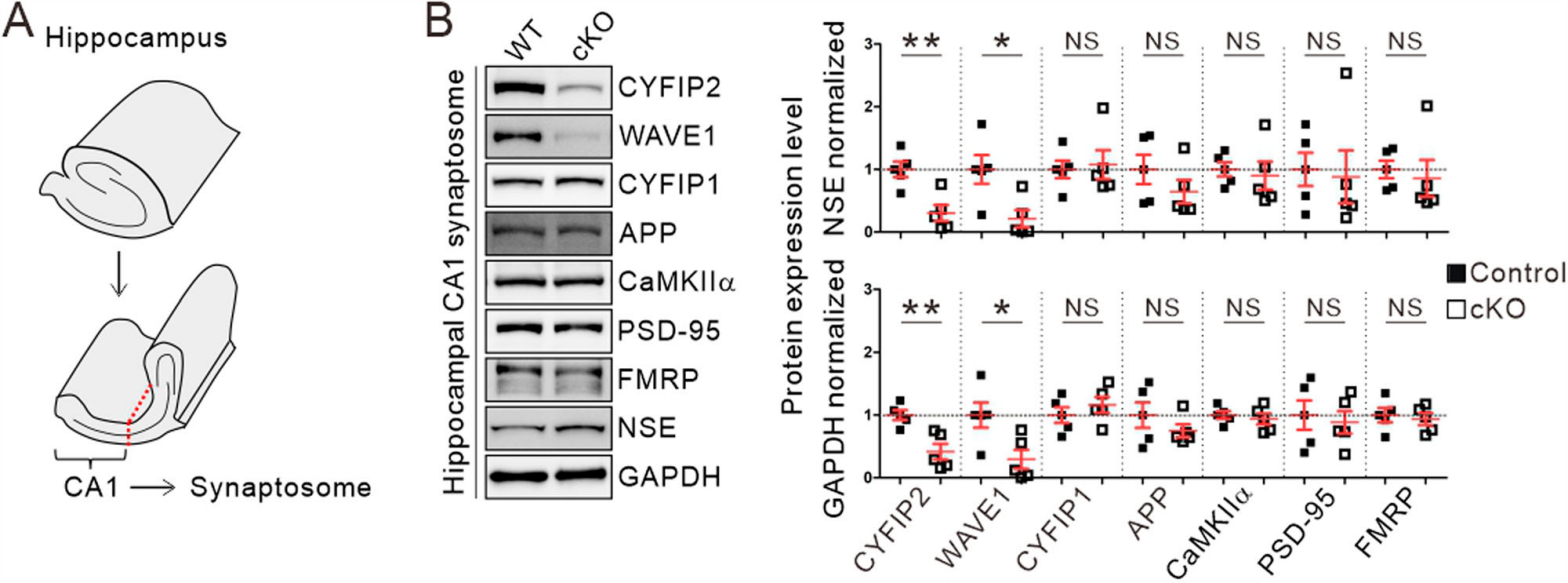
Normal synaptosomal expression levels of AD-related proteins, APP and CaMKIIα, in the hippocampal CA1 region of aged *Cyfip2* cKO mice. (A) Schematic diagram showing the dissection of the CA1 region of the mouse hippocampus. (B) Representative immunoblot images and quantification of the expression levels of CYFIP2, WAVE1, CYFIP1, APP, CaMKIIα, PSD-95, and FMRP proteins in CA1 synaptosomal fraction of aged *Cyfip2* cKO mice compare to control mice. Protein levels were normalized by either a neuron-specific protein, neuron-specific enolase (NSE), or GAPDH. NS, not significant. *N* = 5 mice.

Overexpression of AD-related proteins, such as APP and CaMKIIα, in the synaptic compartment due to aberrant local mRNA translation, has been proposed as a molecular mechanism underlying AD-like pathologies in *Cyfip2* het mice (Tiwari et al. 2016; Ghosh et al. 2020). Therefore, we analyzed the protein levels of APP and CaMKIIα in the hippocampus of 12-month-old control and *Cyfip2* cKO mice. We prepared a crude synaptosomal fraction from the dissected hippocampal CA1 region and performed immunoblotting (Figure 3(A)). Consistent with the immunohistological analysis, CYFIP2 levels were reduced in hippocampal CA1 synaptosomal lysates from *Cyfip2* cKO mice compared to control mice (Figure 3(B)). As expected, the WAVE1 protein, another component of the WRC, was also reduced in *Cyfip2* cKO lysates because the stability of WAVE1 is inter-dependent with that of CYFIP2 (Han et al. 2015; Zhang et al. 2020; Kang et al. 2023). In contrast, CYFIP1 levels were comparable between control and *Cyfip2* cKO mice. Notably, we found that neither APP nor CaMKIIα protein levels were significantly altered in *Cyfip2* cKO mice compared to control mice (Figure 3(B)). Furthermore, the levels of PSD-95, another synaptic protein whose mRNA stability and translation are regulated by FMRP (Zalfa et al. 2007), and FMRP itself were normal in the CA1 synaptosome of aged *Cyfip2* cKO mice. Taken together, these results suggest that there is no overt AD-like pathology in the hippocampal CA1 region of 12-month-old *Cyfip2* cKO mice.

## Discussion

In this study, we combined immunohistochemical, morphological, and biochemical approaches to understand whether the cell-autonomous reduction of CYFIP2 in excitatory pyramidal neurons is sufficient to induce AD-like pathologies in the hippocampal CA1 region. However, none of the results showed a significant AD-like phenotype in aged *Cyfip2* cKO mice, in contrast to the severe phenotypes observed in *Cyfip2* het mice (Tiwari et al. 2016; Ghosh et al. 2020). Therefore, our results suggest that other neurons and/or their synaptic connections with CA1 pyramidal neurons are also critically involved in the hippocampal AD-like phenotypes of *Cyfip2* het mice. Additional genetic or viral tools to reduce CYFIP2 protein levels in specific or combinatorial neurons of the hippocampal circuit will help us further address this issue.

**Figure 4.**
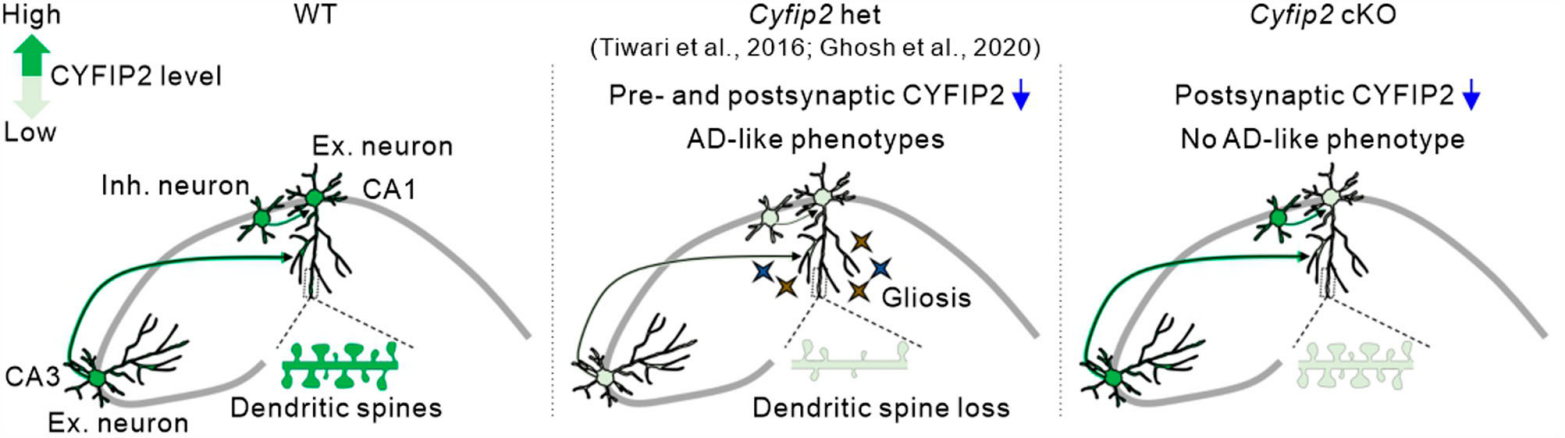
Schematic diagrams summarizing the different hippocampal phenotypes among aged wild-type (WT), *Cyfip2* het, and *Cyfip2* cKO mice. CYFIP2 protein levels are reduced in both CA1 and CA3 excitatory and inhibitory neurons in *Cyfip2* het mice, but they are only reduced in CA1 excitatory neurons in *Cyfip2* cKO mice. In the hippocampal CA1 region, AD-like pathologies, such as gliosis and dendritic spine loss, are observed in *Cyfip2* het mice. In contrast, no AD-like phenotype is observed in *Cyfip2* cKO mice. Ex., excitatory; Inh., inhibitory.

Based on previous findings, we speculate on some mechanisms that explain the lack of an AD-like phenotype in *Cyfip2* cKO mice (Figure 4). CYFIP2 mRNAs and proteins are predominantly expressed in neurons compared to non-neuronal cells in the brain and are detected in both excitatory and local inhibitory neurons (Zhang, Kang, et al. 2019b; Lee SH et al. 2020; Ma et al. 2022). Moreover, CYFIP2 is expressed in neurons of the hippocampal CA3 region as well as of other brain regions that can directly form synaptic connections with CA1 excitatory pyramidal neurons (Han et al. 2015; Lee SH et al. 2020). As CYFIP2 regulates axonal and presynaptic development and function (Cioni et al. 2018; Kim GH et al. 2020), it is conceivable that both presynaptic and postsynaptic compartments are functionally affected in CA1 pyramidal neurons of *Cyfip2* het mice, thereby ultimately leading to synaptic loss. Meanwhile, the postsynapse-specific CYFIP2 reduction in CA1 pyramidal neurons of *Cyfip2* cKO mice may be insufficient to induce such changes. Furthemore, considering the concept of trans-synaptic propagation of pathology in AD (Tzioras et al. 2023), bi-directional spread of AD pathology through synaptic connections with other abnormal neurons may synergistically worsen the phenotypes of CA1 pyramidal neurons of *Cyfip2* het mice compared to those of *Cyfip2* cKO mice.

At the molecular level, local mRNA translation of AD-related proteins by the CYFIP2-FMRP-eIF4E complex can be differentially affected in the CA1 pyramidal neurons of *Cyfip2* het and *Cyfip2* cKO mice (Ghosh et al. 2020). Indeed, we observed normal synaptosomal levels of APP and CaMKIIα proteins in the hippocampal CA1 of *Cyfip2* cKO mice, unlike their increased levels in *Cyfip2* het mice (Tiwari et al. 2016). The interaction of CYFIP1 with FMRP and eIF4E is regulated by synaptic activity (De Rubeis et al. 2013). Therefore, it can be speculated that even with CYFIP2 reduction, relatively normal synaptic functions in neurons of *Cyfip2* cKO mice could preserve local mRNA translation within the normal range. However, in *Cyfip2* het mice, more profound changes in synaptic activity, due to both presynaptic and postsynaptic reduction of CYFIP2, and changes in the CYFIP2-FMRP-eIF4E complex may congruently lead to aberrant mRNA translation and overproduction of AD-related proteins. Further investigations of the molecular composition and function of the CYFIP2-FMRP-eIF4E complex in *Cyfip2* het and *Cyfip2* cKO neurons are needed to test this hypothesis.

Considering the aforementioned speculations regarding the mechanisms involved, another plausible explanation for the observed phenotypic difference between *Cyfip2* het and *Cyfip2* cKO CA1 pyramidal neurons could be the delayed onset of disease in *Cyfip2* cKO mice compared to *Cyfip2* het mice. Due to the preservation of normal presynaptic inputs in CA1 pyramidal neurons of *Cyfip2* cKO mice, it is possible that the neurons will take a longer time to exhibit AD-like phenotypes compared to the neurons of *Cyfip2* het mice. This hypothesis can be examined by investigating the hippocampus of older (e.g. 18-month-old) *Cyfip2* cKO mice and age-matched control mice.

We observed a reduced number of thin, but normal stubby and mushroom spines in the basal dendrites of CA1 pyramidal neurons of aged *Cyfip2* cKO mice. However, there was no significant change in the number of thin, stubby, and mushroom spines in the apical dendrites. The basal dendrite-specific decrease in dendritic spine number was also observed in the layer 5 neurons of the mPFC of young adult *Cyfip2* cKO mice (Zhang et al. 2020), although the underlying mechanism remains unknown. Additionally, a previous study found that overexpression of CYFIP2 increases excitatory synapse number in cultured hippocampal neurons at 14 days in vitro (Davenport et al. 2019), suggesting that CYFIP2 dosage may be an important factor regulating the development and maintenance of excitatory synapses in hippocampal neurons. Our results also indicate that regulation of the F-actin dynamics via the WRC may be a key mechanism, as we observed changes in F-actin levels and downregulation of WAVE1 in the hippocampal CA1 region of *Cyfip2* cKO mice.

In conclusion, our results suggest that excitatory neuron-specific reduction of CYFIP2 is insufficient to induce AD-like pathologies in the hippocampal CA1 region, warranting further investigation into the neuronal circuit-dependent mechanisms of AD pathology induced by CYFIP2 reduction. Beyond identifying the pathogenic mechanisms of AD, these investigations will potentially provide critical neuronal targets in the brain for novel therapeutic approaches to AD.

## Supplementary Material

Supplemental Material
