# Supplementary material for: Cell-autonomous reduction of CYFIP2 is insufficient to induce Alzheimer's disease-like pathologies in the hippocampal CA1 pyramidal neurons of aged mice
Source: Anim Cells Syst (Seoul). 2023 Mar 24;27(1):93–101. doi: 10.1080/19768354.2023.2192263 (PMC10044167; doi:10.1080/19768354.2023.2192263)
Supplement: Supplemental Material [file TACS_A_2192263_SM2820.docx]

**Supporting online material**

**Cell-autonomous reduction of CYFIP2 is insufficient to induce Alzheimer’s disease-like pathologies in the hippocampal CA1 pyramidal neurons of aged mice**

**Materials and Methods**

***PCR primers***

The primer sequences used to genotyping PCR are as follows (Figure 1C):

Forward primer a 5’-ccttcttcatttcctgccata-3’

Reverse primer b 5’-agggctgaggtcaagcaga-3’

Reverse primer c 5’-gaccatgcaatgccttatga-3’

***Brain lysate preparation and immunoblotting***

Brain tissue was homogenized in buffered sucrose (0.32 M sucrose, 4 mM HEPES, 1 mM MgCl_2_, 0.5 mM CaCl_2_, pH 7.3) with freshly added protease and phosphatase inhibitors (Sigma-Aldrich, St. Louis, MO, USA, #05892970001 and #04906837001, respectively). The homogenate was centrifuged at 900 *× g* for 10 min. The resulting supernatant was centrifuged at 12,000 *× g* for 15 min. The pellet was resuspended in buffered sucrose and centrifuged at 13,000 *× g* for 15 min, and the resulting pellet is called P2 (synaptosomal fraction). For each sample, 10–20 μg of protein was loaded onto 4–15% Mini-PROTEAN TGX™ Precast Protein Gels (Bio-Rad, #4561084) for immunoblotting. Proteins were then transferred to a nitrocellulose membrane (GE Healthcare, #10600001). The primary antibodies used for immunoblot analysis were APP (Sigma-Aldrich, #MABN10), CaMKIIα (Thermo Scientific, #MA 1-048), CYFIP1 (Sigma-Aldrich, #AB6046; Millipore, #07-531), CYFIP2 (Abcam, #ab95969), FMRP (Cell Signaling Technology, # 4317), GAPDH (Cell Signaling Technology, #2118), neuron-specific enolase (NSE, Millipore, #AB951-I), PSD-95 (Cell Signaling Technology, # 2507), and WAVE1 (Abcam, # ab75048). Western blot images were acquired using the ChemiDoc^TM^ Touch Imaging System (Bio-Rad) and quantified using ImageJ software.

***Statistical analysis***

All quantifications were performed by researchers who were blinded to the genotype and did not participate in immunostaining/blotting or image acquisition processes. *P* values were calculated using a two-tailed Student’s t-test performed with GraphPad Prism 5 software. All data are presented as mean ± standard error of the mean (SEM). **P* < 0.05; ***P* < 0.01; ∗∗∗*P* < 0.001.
